# Supplementary material for: Theaflavin-Enriched Fraction Stimulates Adipogenesis in Human Subcutaneous Fat Cells
Source: Int J Mol Sci. 2019 Apr 25;20(8):2034. doi: 10.3390/ijms20082034 (PMC6515531; doi:10.3390/ijms20082034)
Supplement: Supplementary file 1 [file ijms-20-02034-s001.pdf]

[Supplemental Information]

The theaflavin-enriched fraction stimulates adipogenesis in human subcutaneous fat cells

Phil June Park, Chan-Su Rha, Sung Tae Kim

Supplemental Figure 1. Chemical structure of (-)-epicatechins and theaflavins. (A) (-)-epicatechin, (B) (-)-epigallocatechin, (C) (-)-epicatechingallate, (D) (-)-epigallocatechin gallate, (E) theaflavin, (F) theaflavin 3,3´-di-*O*-gallate.

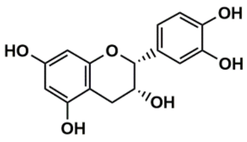

(A)

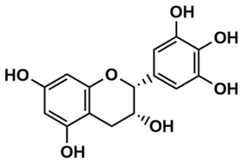

(B)

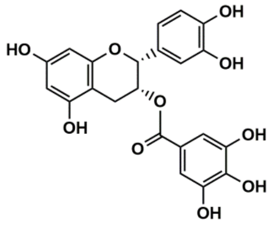

(C)

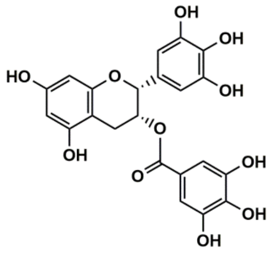

(D)

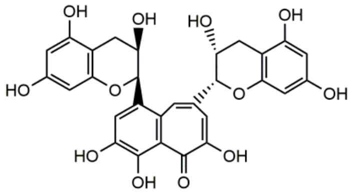

(E)

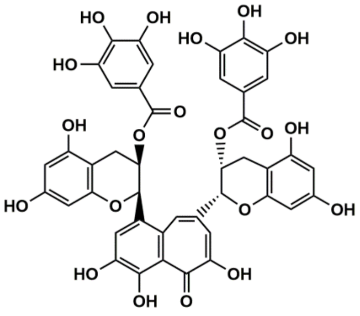

(F)

Supplemental Figure 2. Preparative chromatography of CoF-GT for obtaining TF-enriched fraction (the 4th fraction).

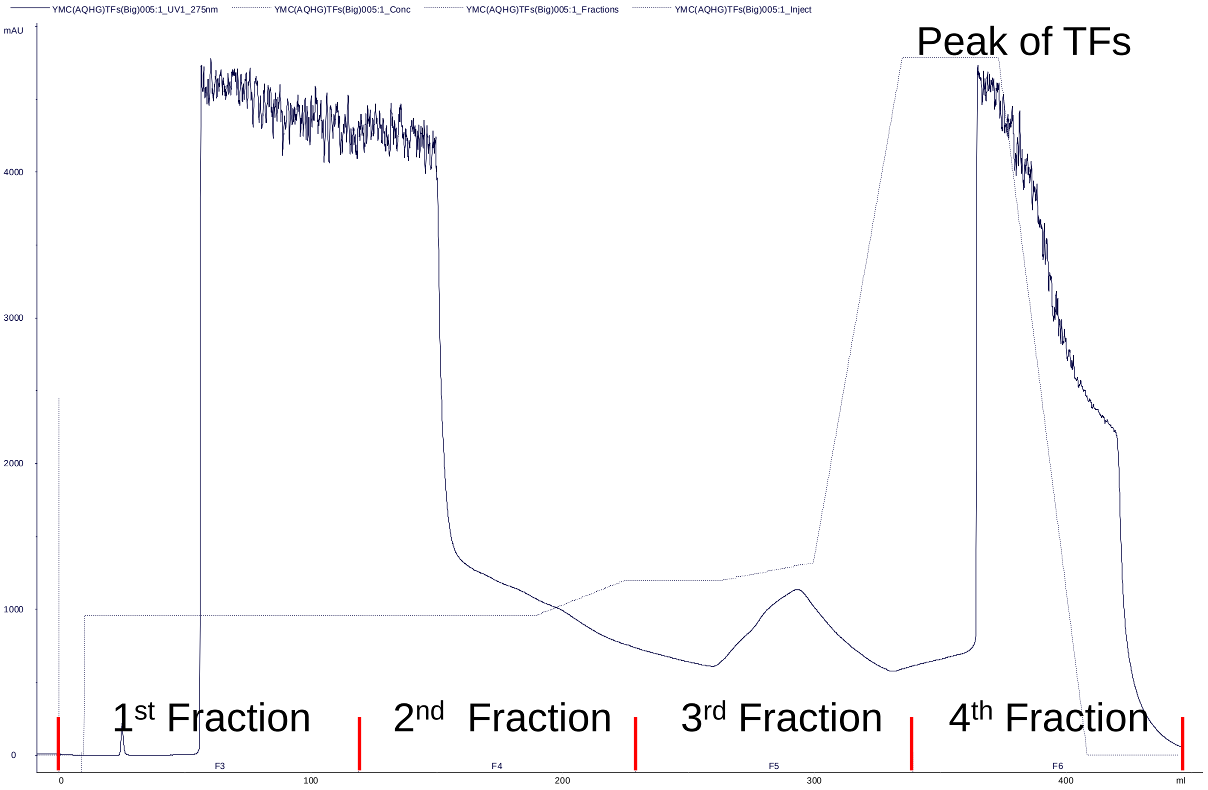

Table 1. The contents of theaflavins in samples.

Sample	TF	TF3G	TF3'G	TFDG	Sum
* CoF-GT rich fraction	21.56±0.96<br>(41.0%)	20.70±0.93<br>(39.4%)	4.11±0.2<br>(7.8%)	6.21±0.22<br>(11.8%)	52.57±2.30<br>(100%)
# F-GT richfraction	7.04±0.29<br>(19.2%)	15.49±0.71<br>(42.3%)	3.18±0.16<br>(8.7%)	10.89±0.52<br>(29.8%)	36.61±1.67<br>(100%)

* Data shown: **theaflavins mg/g of fraction of extract** (mean ± SEM, n=3)

Lower data shown: theaflavins % (w/w) of fraction of extract (mean, n=3)
